# The Cytoplasmic Domain of MUC1 Induces Hyperplasia in the Mammary Gland and Correlates with Nuclear Accumulation of β-Catenin

**DOI:** 10.1371/journal.pone.0019102

**Published:** 2011-04-20

**Authors:** Yuan Li, Haiying Yi, Yixin Yao, Xiaodong Liao, Yiqun Xie, Jie Yang, Zheng Yan, Long Wang, Shunyuan Lu, Ying Kuang, Mingmin Gu, Jian Fei, Zhugang Wang, Lei Huang

**Affiliations:** 1 Department of Medical Genetics, E-Institutes of Shanghai Universities, Shanghai Jiao Tong University School of Medicine, Shanghai, P.R. China; 2 Department of Breast Surgery, Shanghai Huangpu Center Hospital, Shanghai, P.R. China; 3 Shanghai Research Centre for Model Organisms, Shanghai, P.R. China; Northwestern University Feinberg School of Medicine, United States of America

## Abstract

MUC1 is an oncoprotein that is overexpressed in up to 90% of breast carcinomas. A previous *in vitro* study by our group demonstrated that the cytoplasmic domain of MUC1 (MUC1-CD), the minimal functional unit of MUC1, contributes to the malignant phenotype in cells by binding directly to β-catenin and protecting β-catenin from GSK3β-induced degradation. To understand the *in vivo* role of MUC1-CD in breast development, we generated a MUC1-CD transgenic mouse model under the control of the MMTV promoter in a C57BL/6J background, which is more resistant to breast tumor. We show that the expression of MUC1-CD in luminal epithelial cells of the mammary gland induced a hyperplasia phenotype characterized by the development of hyper-branching and extensive lobuloalveoli in transgenic mice. In addition to this hyperplasia, there was a marked increase in cellular proliferation in the mouse mammary gland. We further show that MUC1-CD induces nuclear localization of β-catenin, which is associated with a significant increase of β-catenin activity, as shown by the elevated expression of cyclin D1 and c-Myc in MMTV-MUC1-CD mice. Consistent with this finding, we observed that overexpression of MUC1-C is associated with β-catenin nuclear localization in tumor tissues and increased expression of Cyclin D1 and c-Myc in breast carcinoma specimens. Collectively, our data indicate a critical role for MUC1-CD in the development of mammary gland preneoplasia and tumorigenesis, suggesting MUC1-CD as a potential target for the diagnosis and chemoprevention of human breast cancer.

## Introduction

The mammary gland is composed of a ductal epithelium and surrounding stroma. Development of the mammary gland is initiated with the formation of a small ductal tree in the embryo but progresses predominantly after birth in defined stages associated with sexual development and reproduction, including prepuberty, puberty, pregnancy, lactation, and involution. The primary mammary tissue remains quiescent until puberty, and upon stimulation of circulating growth hormone and estrogen, the distal end of the duct enlarges to form a bulb-like terminal end bud (TEB), which is a highly proliferative epithelial structure. TEBs drive the epithelial ductal tree to elongate and branch rapidly, leading to the formation of an extended ductal system that fills the entire fat pad. The ducts branch into smaller ductules and develop alveoli during pregnancy, which then differentiate into secretory epithelial cells during parturition. The ductal system is surrounded by a continuous periductal stroma that consists of fibroblasts, macrophages, eosinophils and vascular cells within the confines of the mammary fat pad [1], [2].

Many genetic pathways have been identified as being involved in mammary development [1]. Among these pathways, Wnt/β-catenin signaling has been documented to have critical functions during mammary bud patterning, its formation during embryogenesis and its elongation during puberty, as well as in the development of milk production during pregnancy [3], [4], [5]. The canonical Wnt or Wnt/β-catenin pathway operates by inhibiting proteolysis of cytoplasmic β-catenin, allowing it to enter the nucleus and regulate gene transcription through the lymphoid enhancer factor/T cell factor (Lef/Tcf) transcription factors, thereby promoting cell proliferation and survival [6]. Both loss-of-function and gain-of-function studies suggest that Wnt signaling is of importance during alveolar development [3], [7]. It may promote ductal side-branching in early pregnancy and may be essential for the proliferation and survival of lobuloalveolar progenitor cells in later pregnancy. In addition to regulating mammary development, the Wnt/β-catenin pathway is also associated with breast cancer. Expression of MMTV-ΔN89β-catenin induces precocious lobuloalveolar development and multiple aggressive adenocarcinomas early in life [8]. Moreover, activation of β-catenin signaling in basal mammary epithelial cells was also found to affect the entire process of mammary gland development and give rise to tumors [9].

The transmembrane protein mucin MUC1 is expressed on most apical surfaces of secretory epithelia, including the mammary gland, as well as some hematopoietic cells. It was initially identified as a human breast tumor antigen because it was aberrantly overexpressed in more than 70% of human carcinomas with a loss of polarity, special in 90% of human breast carcinomas [10], [11]. Overexpression of MUC1 has been shown to confer anchorage-independent growth and tumorigenicity in cells [12], [13]. Studies using mouse models have further established roles for MUC1 in the promotion and invasiveness of breast cancer. Overexpression of human MUC1 in MMTV-MUC1 transgenic mice indicated the ability of MUC1 to promote in vivo transformation of the mammary gland by forming a complex with β-catenin and potentiating EGF-dependent activation of MAP kinase signaling pathways, thereby inhibiting normal glandular involution[14], [15]. A recent study also showed the ability of Muc1 to control the development and tumorigenesis of myeloid-derived suppressor cells (MDSCs) [16]. However, a deficiency of Muc1 results in both reduced tumor growth and spreading in MMTV1-mTag mice [17].

The human MUC1 gene spans 4 to 7 kb [18]. Following translation as a large precursor polypeptide, it is cleaved into N- and C-terminal subunits in the endoplasmic reticulum, and the two subunits form a stable noncovalent complex at the cell membrane. The MUC1 NH2-terminal subunit (MUC1-N) consists of variable numbers of highly glycosylated 20-amino-acid tandem repeats (>250 kDa) [19] that contribute to a physical barrier protecting epithelial cells from damage due to external environmental exposure. The MUC1 COOH-terminal subunit (MUC1-C) is composed of a 58-amino-acid extracellular domain, a 28-amino-acid transmembrane domain, and a 72-amino-acid cytoplasmic domain. Glycosylation on Asn36 of the MUC1-C extracellular domain induces MUC1 to bind to the galectin-3 ligand and physically associate with the epidermal growth factor receptor (EGFR) [15], [20] and other receptor tyrosine kinases. The MUC1-C cytoplasmic domain (MUC1-CD) contains seven Tyr residues, five of which can act as binding motifs for proteins with SH2 domains when phosphorylated. Moreover, the MUC1-CD contains a serine-rich motif (SRM) with homology to sequences in E-cadherin and APC protein that function as β-catenin binding sites [21]. GSK3β phosphorylates MUC1-CD on a serine in an SPY site that is just upstream of the SRM [22]. The phosphorylation of MUC1-CD by GSK3β decreases the interaction with β-catenin [22]. Conversely, both src and erbB receptor tyrosine kinases phosphorylate MUC1, as well as the serine/threonine kinase protein kinase C delta (PKCδ), and these phosphorylation events promote interactions between β-catenin and MUC1 [15], [23], [24]. Importantly, MUC1-C is detectable in the nucleus [25], [26] and functions as a coactivator of β-catenin-Tcf–mediated transcription in cells [27].

Our previous studies showed that MUC1-CD binds directly to β-catenin and blocks the GSK3β-mediated phosphorylation and degradation of β-catenin, thereby contributing to the malignant phenotype in cells [12]. However, the role of MUC1-CD in mammary development has only been partially elucidated. In the current study, we generated a mouse mammary tumor virus (MMTV)-MUC1-CD transgenic mouse model and investigated the function of MUC1-CD in mammary development in a C57BL/6J background mouse that is more resistant to breast tumorigenesis.

## Results

### MUC1-CD is expressed in luminal epithelial cells of the mammary gland

**Figure 1:**
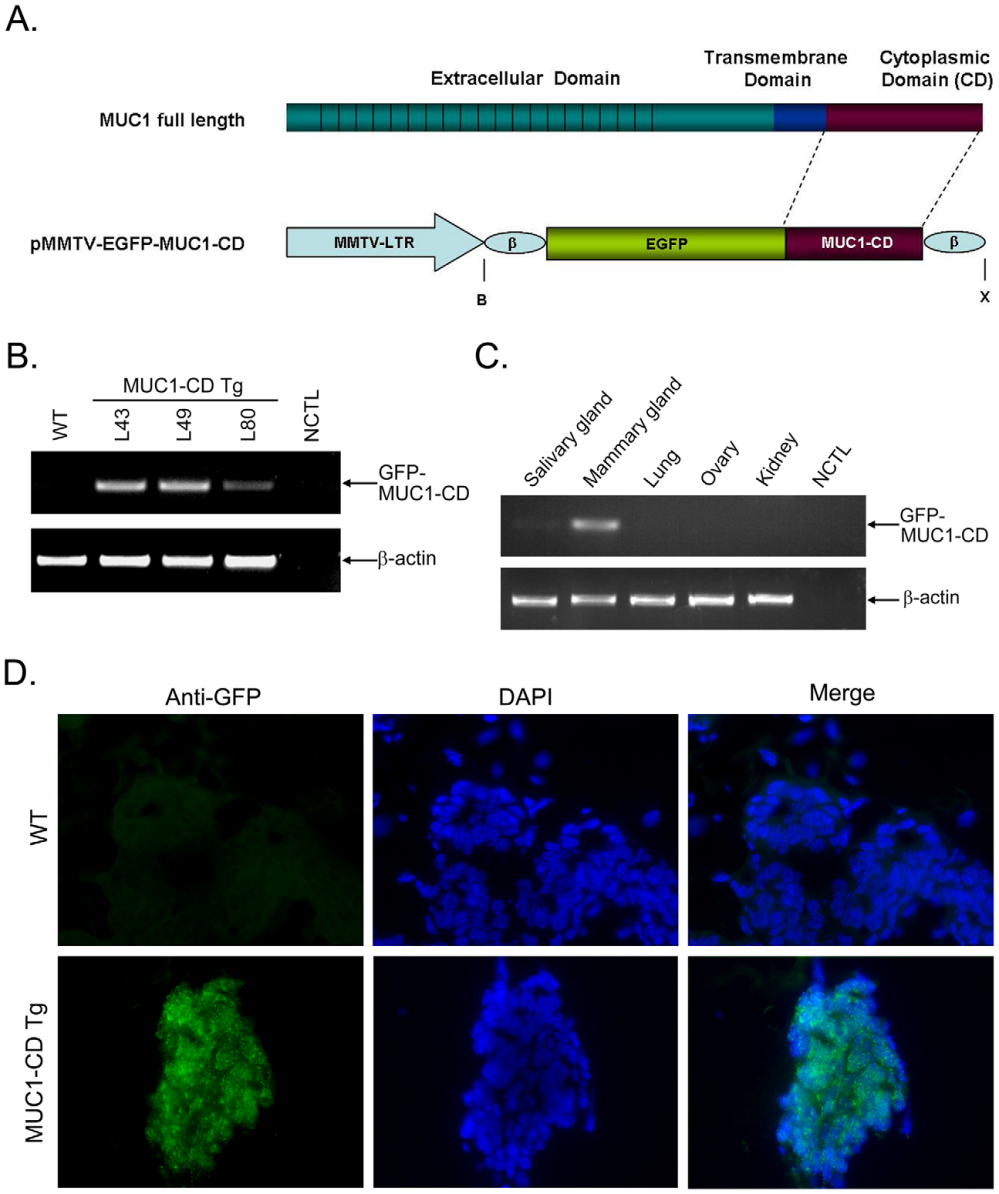
Generation of MMTV-MUC1-CD transgenic mice. (A) Schematic representation of the transgene construct. MMTV-LTR, mouse mammary tumor virus long terminal repeat; β, β-globin intron/exon/poly A sequences; B, BamHI; X, XhoI. (B) RT-PCR analysis of GFP-MUC1-CD and β-actin expression in day 13.5 pregnant mouse mammary glands. WT, Wild-type; L43, L49, and L80 indicate cDNA from Lines 43, 49 and 80, respectively; NCTL, negative control, PCR without template. (C) RT-PCR analysis of GFP-MUC1-CD and β-actin expression in the indicated organs of an L43 adult female mouse. (D) IF staining of GFP performed on frozen sections of wild-type (upper panel) and transgenic mouse mammary glands (lower panel) at day 13.5 of pregnancy. Nuclei were stained with DAPI. The slides were analyzed by flourescence microscopy. Similar results were obtained with the separately mice. Photomicrographs obtained at a 400× magnification.

To investigate the role of MUC1-CD in mammary development, we generated transgenic mice that overexpress GFP-tagged human MUC1-CD (72 AA) cDNA under the control of an MMTV promoter [28],[29] in the mammary gland with the construct shown in Fig. 1A. Four founders with genomic integration of the transgene were obtained by PCR analysis of tail gDNA from potential MMTV-GFP-MUC1-CD (MUC1-CD) founder transgenic mice (data not show). Three of these individuals (L43,L49 and L80) displayed Mendelian transmission to the F1 generation and expressed the transgene, as determined by RT-PCR using total RNA (Fig. 1B). The band is not found in wild-type littermates or negative controls that did not contain the template (Fig. 1B). To test the tissue specificity of transgene expression, RT-PCR was further performed using total RNA from the mammary gland, salivary gland, lung, ovary and kidney from a L43 virgin 8w female transgenic mouse [30]. The results showed that MUC1-CD was detectable in the mammary gland and weakly expressed in the salivary gland. Little, if any, expression was detected in the lung, ovary and kidney (Fig. 1C). To determine the distribution of MUC1-CD, immunofluorescence staining for anti-GFP in the frozen sections was carried out in the mammary glands at pregnancy day 13.5 of MUC1-CD transgenic mice and wild-type littermates. The results indicated that the GFP-MUC1-CD was expressed in the cytoplasm and nuclei of epithelial cells in transgenic mouse mammary ducts, and no expression was detectable in wild-type littermates (Fig. 1D).

### MUC1-CD induces mammary hyperplasia in transgenic mice

**Figure 2:**
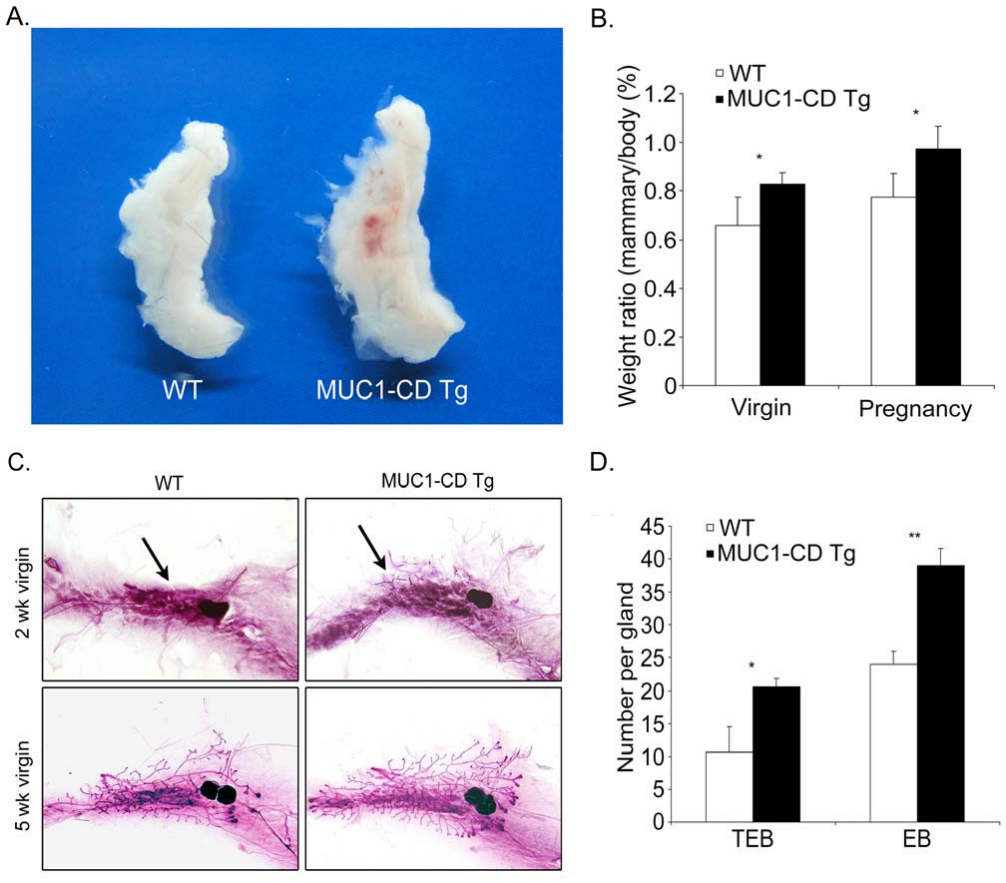
MUC1-CD induces precocious development of mammary glands during prepuberty and early puberty. (A) Mammary glands from a MUC1-CD transgenic female mouse (right) and a wild-type littermate (left). (B) Comparison of the weight/body ratio of the fourth mammary glands between MUC1-CD transgenic mice (n = 4) and wild-type littermates (n = 4). (C) Carmine alum staining of whole mounts of inguinal mammary glands from a wild-type female (left) and transgenic female (right) in 2-wk-old virgins (upper) and 5-wk-old virgins (lower). As indicated by the arrows, in 2-wk-old virgins, the precocious development of mammary epithelial ducts was apparently observed in transgenic mice (upper right) compared to wild-type littermates (upper left). (D) Quantification of the number of terminal end buds (TEBs) and end buds (EBs) in MUC1-CD transgenic mice (n = 3) and wild-type littermates (n = 3) at 5 wks of age. *P<0.05; **P<0.01.

To determine whether MUC1-CD has effects on mammary development, the inguinal mammary glands from four mice of 8-wk-old virgin females for each group were excised and compared between MMTV-GFP-MUC1-CD transgenic mice and wild-type littermates. The mammary glands of the transgenic mice appeared larger in volume than that of wild-type littermates (Fig. 2A). A statistically significant difference (p<0.05) was found when we compared the weight/body ratio of the inguinal mammary gland between transgenic mice and wild-type littermates in 8-wk-old virgins and pregnant females (Fig. 2B). To investigate this in more detail, we examined whole-mount preparations of the inguinal mammary glands from early virgin females of MUC1-CD transgenic mice and wild-type littermates. In 2-wk-old virgins, precocious development of mammary epithelial ducts was observed in transgenic mice compared with wildtype littermates (Fig. 2C upper). Marked differences were seen in 5-wk-old virgin transgenic and wild-type mice. Wild-type mammary ducts extended to the lymph node, while transgenic mammary ducts extended beyond the lymph node and exhibited an extent of elongation (Fig. 2C lower). In addition, transgenic mammary tissue displayed a nearly 100% increase in terminal end buds (TEBs) and a 80% increase in end bud (EB) structures compared to wild-type littermates (Fig. 2D).

**Figure 3:**
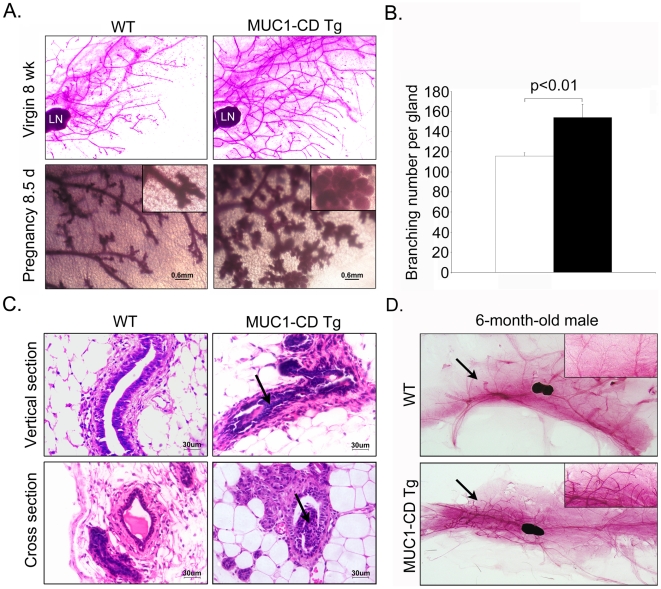
MUC1-CD induces a lobular hyperplasia phenotype in the mammary gland. (A) Whole mounts of inguinal mammary glands from wild-type (left) and transgenic (right) mice at 8 wks (upper panel) and pregnancy day 8.5 (lower). Insets in each panel are higher magnifications (200X), scale bars, 0.6 mm, LN: lymph node. (B) Branch-point analysis in MUC1-CD transgenic mice (n = 3) and wild-type littermates (n = 3) at 8 wks of age. P<0.01. (C) H&E staining of sections from mammary glands of wild-type (left) and transgenic (right) mice at pregnancy day 8.5. As indicated by the arrow, cells filled the ducts of transgenic mammary glands. All images were acquired at the same magnification; scale bars, 30 µm. (D) Representative images of inguinal mammary glands whole mounts from wild-type (upper) and transgenic (lower) male mice at 6 months. Insets in each panel are higher magnifications.

The mammary glands from 8-wk-old virgins were further examined at higher magnifications. The mammary ductal tree from the transgenic mice displayed a more complex structure than that from wild-type mice (Fig. 3A upper panel). In addition, branch-point analysis indicated a statistically significant increase in branching in transgenic mice (Fig. 3B). Transgenic mammary glands also showed precocious development of lobuloalveoli in females at 8.5 days of pregnancy compared with stage-matched wild-type littermates (Fig. 3A lower panel). The results suggest that MUC1-CD is sufficient for promoting ductal branching and the formation of alveolar lobules. To further investigate the structure of mammary ducts, hematoxylin and eosin (H&E) staining was performed on paraffin sections from females at 8.5 days of pregnancy, and a large number of cells were found in the mammary gland lumen of transgenic mice but not in wild-type littermates, indicating a luminal epithelium hyperplasia phenotype in the transgenic mice (Fig. 3C). To determine whether MUC1-CD also effect on mammary gland of male mice, which without ovarian hormonal function, we analyzed 3 pairs of whole-mount preparations of the inguinal mammary glands from 6-month-old male and 20-month-old male mice. We found that transgenic mice showed a hyperbranch phenotype when compared with their wild-type littermates (Fig. 3D). These data demonstrate that expression of MUC1-CD induces mammary gland hyper-branching in both of female and male mice and extensive lobulo-alveolar development in female, these are associated with a hyperplasia phenotype.

### MUC1-CD promotes mammary cellular proliferation

**Figure 4:**
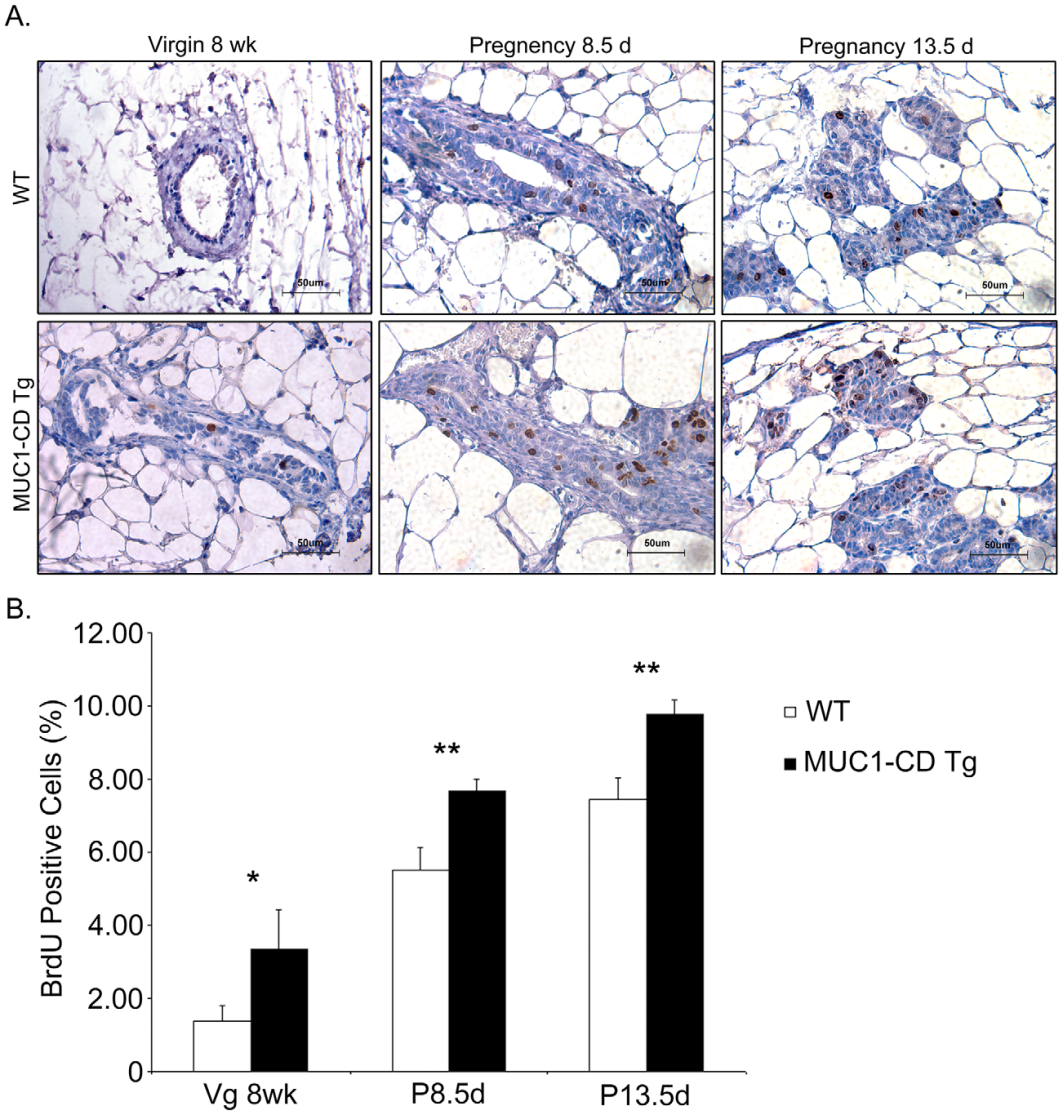
MUC1-CD promotes cellular proliferation of the mammary gland. (A) Representative images of BrdU assays. BrdU was administered intraperitoneally to mice at 8 wks, 8.5 days and 13.5 days of pregnancy 2 h before sacrifice. Incorporated BrdU was detected in sections by IHC using an anti-BrdU antibody. Magnification: 400X. (B) The number of BrdU-positive epithelial cells in mammary lumina at 8 wks, pregnancy day 8.5 and pregnancy day 13.5 were counted. For each sample, cells were counted in 10 random high-power fields. Three mice were analyzed per group. The results are expressed as the percentage (mean±S.D.) of BrdU-positive cells. *P<0.05; **P<0.01; scale bars, 50 µm.

In light of the finding that MUC1-CD induces the development of mammary hyper-branching and extensive lobuloalveoli, we asked whether MUC1-CD expression was associated with enhanced cellular proliferation or reduced apoptosis. Measurement of mammary cell proliferation was performed based on BrdU incorporation in 8-wk-old virgins and females at day 8.5 and 13.5 of pregnancy. The results revealed that there were more BrdU-positive epithelial cells in the mammary gland lumina of MUC1-CD transgenic mice than that in wild-type mice at all three stages (Fig. 4A). Quantitative analysis indicated that statistically significant differences were found for the number of BrdU-positive cells between MUC1-CD transgenic mice and wild-type littermates at all three stages (virgin 8 week: 3.3±1.1% versus 1.4±0.4%, P = 0.045; 8.5 days pregnant: 7.7±0.3% versus 5.5±0.6%, P = 0.006; 13.5 days pregnant: 9.8±0.4% versus 7.4±0.6%, P = 0.005) (Fig. 4B). However, TUNEL staining assays at these three stages showed no detectable difference between transgenic mice and wild-type littermates (data not shown). These data demonstrate that overexpression of MUC1-CD promotes cellular proliferation in the mammary glands of transgenic mice.

### MUC1-CD increases β-catenin nuclear localization and activity in the mouse mammary gland

**Figure 5:**
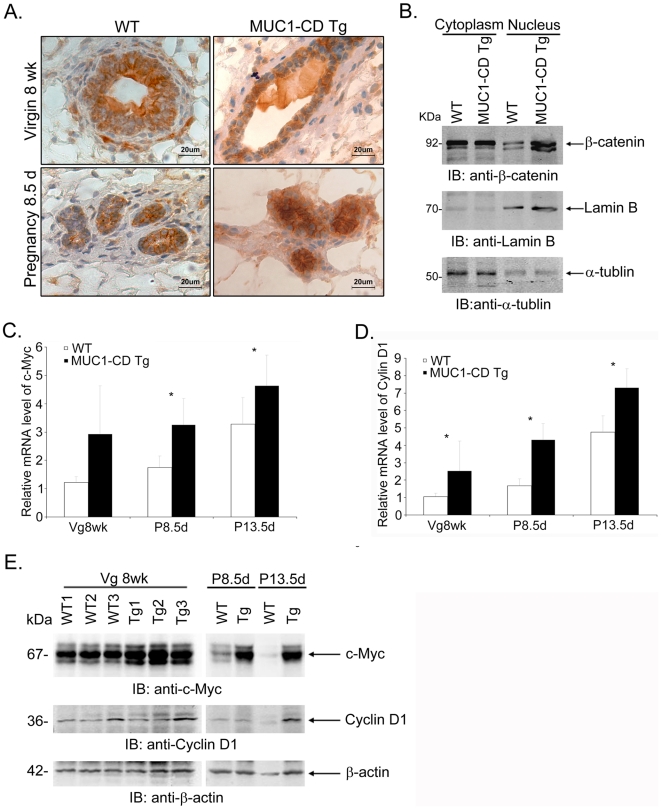
MUC1-CD increases β-catenin nuclear localization and activity in the transgenic mice. (A) IHC staining of β-catenin at 8 wks and pregnancy day 8.5. Scale bars, 20 µm. (B) Nuclear and cytoplasmic fractions of the mammary glands from pregnancy day 13.5 females were immunoblotted with antibodies against β-catenin, lamin B, and α-tubulin. Quantitative RT-PCR analysis of c-Myc (C) and cyclin D1 (D) mRNA levels in 8-wk-old virgin, pregnancy day 8.5 and pregnancy day 13.5 mouse mammary glands. Results are shown as mean±S.D. (n≥3 for each genotype at each time point). *P<0.05. (E) Lysates from the mammary gland of 8-wk-old virgin, pregnancy day 8.5 and pregnancy day 13.5 mice were immunoblotted with the indicated antibodies.

Previous studies have demonstrated that MUC1-CD overexpression promotes cell proliferation in vitro via stabilization of β-catenin [12]. To dissect the mechanism underlying the effect of MUC1-CD on mammary cellular proliferation in vivo, we investigated the expression of β-catenin in 8-wk-old virgin and females at day 8.5 of pregnancy by IHC staining. The results demonstrated a clear expression of β-catenin in both MUC1-CD transgenic mice and wild-type mice, but the abundance of β-catenin protein was found to be much higher in MUC1-CD transgenic mice than in wild-type littermates (Fig. 5A). In addition, β-catenin was located predominantly in the nucleus at both of these stages in MUC1-CD transgenic mice (Fig. 5A right panel). In contrast, in wild-type mice, it was located mainly in the cytosol and cell membrane. In support of this finding, subcellular fractionation experiments in females at day 13.5 of pregnancy showed that the β-catenin level was increased in the nucleus in MUC1-CD transgenic mice compared to wild-type mice (Fig. 5B). A similar result was obtained for 8-wk-old virgin mice (Data not shown). Cyclin D1 and c-Myc are two important transcriptional targets of the Wnt/β-catenin pathway [31], [32], [33]. To further elucidate the activity of β-catenin, we examined the expression of these two genes in mammary tissues in 8-wk-old virgins and females at day 8.5 and 13.5 of pregnancy. Consistent with the increased activity of β-catenin, MUC1-CD transgenic mice displayed markedly increased mRNA levels of c-Myc, as well as elevated levels of cyclin D1 (Fig. 5C and D). These results were further confirmed by the elevated protein levels of c-Myc and cyclin D1 in MUC1-CD transgenic mice (Fig. 5E). Taken together, these data indicate that MUC1-CD induces the nuclear distribution of β-catenin and subsequent upregulation of c-Myc and cyclin D1 gene expression in the mouse mammary gland.

### MUC1-C is associated with increased β-catenin nuclear localization and activity in clinical breast carcinomas

**Figure 6:**
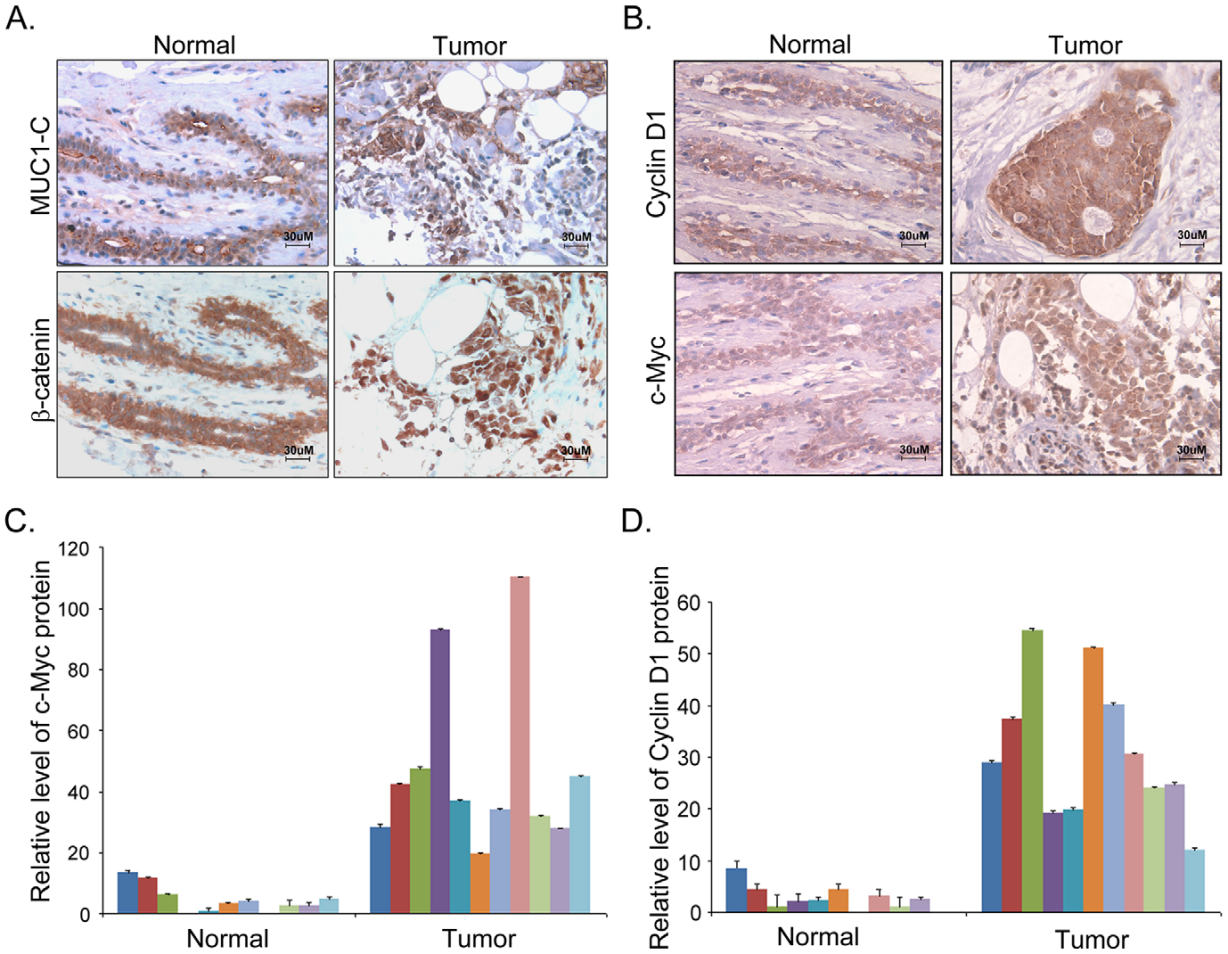
MUC1-C is associated with increased β-catenin nuclear localization and activity in clinical breast carcinomas. (A) Representative images of IHC staining of MUC1-C and β-catenin in sections from human breast cancer tissue and normal mammary tissues. (B) Representative images of IHC staining of Cyclin D1 and c-Myc in sections from human breast cancer tissues and normal mammary tissues. Intensity analysis of c-Myc (C) and Cyclin D1 (D) in sections from 11 human breast cancer tissue samples and 11 normal mammary tissue samples. The data represented as relative fold of intensity of IHC staining in graphs were measured using Image-Pro Plus software; scale bars, 30 µm.

To explore the relationship between MUC1-C expression and β-catenin activity in human breast cancers, we conducted IHC analysis of MUC1-C, β-catenin, c-Myc and Cyclin D1 in human breast cancers and normal tissues. In the normal mammary gland tissues, MUC1-C was expressed weakly on the apical surface of glandular epithelia (Fig. 6A upper left), while β-catenin was mainly localized to the adherens junctions of mammary epithelium cells (Fig. 6A lower left). In contrast, in the human breast cancer specimens, MUC1-C was aberrantly overexpressed in the cytosol and nuclei (Fig. 6A upper right). Additionally, significantly more β-catenin was localized to the nucleus in breast cancer specimens (Fig. 6A lower right). Consistent with this result, Cyclin D1 and c-Myc were expressed at considerably higher levels in tumors compared with normal mammary tissues (Fig. 6B). This result was further confirmed by statistical analysis comparing 11 breast tumor tissues and 11 normal tissues (Fig. 6C & 6D). These data indicate that a high abundance of MUC1-C correlates with an elevated level of β-catenin in the nucleus and upregulation of c-Myc and Cyclin D1 gene expression in human breast carcinomas.

## Discussion

In a published study, transgenic lines overexpressing full-length human MUC1, tail-deleted cytoplasmic MUC1 (MUC1ΔCT), or tandem repeat-deleted MUC1 under the control of an MMTV promoter were generated in an FVB mouse background [15]. Primary mammary gland tumors were observed in the MMTV-MUC1 lines but not in either the MMTV-MUC1ΔCT transgenic line or the wild-type control lines, implicating a function of MUC1-C in tumorigenicity in mammary tissue. In agreement with this notion, MUC1-CD was found to be sufficient for inducing anchorage-independent growth and tumorigenicity in cells, indicating that the MUC1-N mucin subunit is dispensable for transformation. In the present study, we generated MMTV-MUC1-CD transgenic mice in a C57BL/6J background. We found that expression of MUC1-CD in luminal epithelial cells of the mammary gland induced a mammary hyperplasia phenotype characterized by hyperbranching in the transgenic mice at prepuberty and early virgin stages. Although IHC analysis showed a hyperplasia phenotype in the transgenic mice, no tumors were observed, indicating that MUC1-CD is not sufficient to cause tumors in the C57BL/6J background. These results suggest that additional genotypic changes are required for cancer development in this context. But this is not surprising given that C57BL/6J mice are less susceptible to mammary tumorigenesis than FVB mouse background [34], [35]. However, the development of hyperplasia indicates that MUC1-CD plays a role in mammary gland development.

The development of the mammary gland is regulated by a balance between proliferation and apoptosis [36], [37]. MUC1-C is involved in diverse signaling pathways that have been linked to tumorigenesis, functioning either in promoting cell proliferation or blocking the induction of apoptosis [38], [39], [40], [41], [42]. In this study, a statistically significant difference was found between MUC1-CD transgenic mice and wild-type littermates at stages of puberty and pregnancy in our analysis of BrdU-positive cells but not when we examined TUNEL-stained cells (data not shown). Our data demonstrate that the MUC1-CD enhances mammary cellular proliferation, rather than reducing apoptosis, at least during stages from prepuberty to pregnancy.

Estrogen receptor is generally believed to have an essential role in development of the mammary gland at puberty stage [1]. Given that MUC1 stabilizes ERα, stimulates ERα-mediated transcription, contribute to E2-mediated growth and survival of breast cancer cells [43], we asked if the effect of MUC-CD on mammary development is related to enhanced ER activity. Our results show that MUC1-CD induces mammary gland hyper-branching in both of male and female prepuberty mice, suggesting that ER has little, if any, effect on hyperplasia phenotype in MUC1-CD transgenic mice.

A number of studies have revealed a role of the Wnt/β-catenin pathway in the initial stages of mammary development [44]. For example, several Wnt genes, including Wnt3a, Wnt6, Wnt10a, and Wnt10b, are sequentially expressed within the developing mammary tissue between E11.25 and E11.5 (40–42 somite stage), suggesting that Wnt/β-catenin pathway signaling is required for the initiation of mammary gland development in embryos [45]. In support of this finding, mice expressing Wnt inhibitors, such as Dickkopf1 (Dkk1) [45], or that are deficient in Lef-1 [46], [47] exhibit defective embryonic mammary development. In light of our finding that MUC1-CD induces a precocious development of mammary gland at as early as 2 wks of age, we hypothesize that the effect of MUC1 in the early stages of embryonic mammary development could be through the Wnt/β-catenin pathway. Both our IHC and nuclear fraction analyses demonstrated an elevated β-catenin level in the nucleus in MUC1-CD transgenic mice, suggesting that induction of mammary hyperplasia during stages from prepuberty to pregnancy by MUC1-CD could be carried out via regulating β-catenin while bypassing the need for hormonal signals. However, multiple lines of evidence have demonstrated roles for β-catenin signaling in alveologenesis but not in ductal side-branching [48]. We observed both ductal hyper-branching and extensive lobuloalveolar development in MUC1-CD transgenic mice, suggesting another mechanism through which MUC1-CD affects ductal side branching, in addition to the β-catenin pathway.

Upregulation of c-Myc or Cyclin D1 has been identified to result from Wnt/β-catenin pathway activation [31], [32], [33]. The Cyclin D1 protein is a cell cycle regulator that functions to promote cellular proliferation and transformation through inhibiting the retinoblastoma pRB protein [49], [50]. It is overexpressed in up to 50% of human mammary ductal carcinomas in breast cancer [51], [52], [53]. The c-Myc is also an oncogene and is overexpressed in at least 15% of breast cancers. A high expression level of c-Myc is significantly associated with a poor prognosis in breast cancer patients [54]. Transgenic mice constitutively expressing either MMTV-cyclin D1 or MMTV-c-Myc develop mammary hyperplasia and mammary carcinomas [55], [56], [57]. Furthermore, transgenic mice overexpressing the Wnt signaling co-receptor LRP6 driven by the MMTV promoter exhibit activation of Wnt/β-catenin signaling, upregulation of cyclin D1 and c-Myc expression, and increased expression of the cell proliferation marker Ki67, thereby triggering mammary hyperplasia [5]. MUC1-CD transgenic mice display markedly increased levels of c-Myc and cyclin D1 in their mammary tissues at virgin and pregnancy stages, suggesting that induction of mammary hyperplasia by MUC1-CD likely occurs through activation of β-catenin and subsequent upregulation of c-Myc and cyclin D1 gene expression in the mouse mammary gland.

The developing mammary gland displays many of the properties associated with tumor progression, such as invasion, reinitiation of cell proliferation, resistance to apoptosis, and angiogenesis. Thus, many of the factors essential for mammary gland development are also associated with cancer [1], [58]. Indeed, in breast carcinoma specimens, we observed that overexpression of MUC1-C was associated with a nuclear distribution of β-catenin in tumor tissues and increased expression of Cyclin D1 and c-Myc. This result is similar to what we observed in MUC1-CD transgenic mice.

In summary, the present investigation provides evidence for the contribution of MUC1-CD to the early development of the mammary gland. Enhanced proliferation of mammary epithelia could be induced through activation of β-catenin and increased expression levels of cyclin D1 and c-Myc. Deregulation of β-catenin by MUC1-CD may also be a critical mechanism promoting breast carcinogenesis. Therefore, finding a role for the MUC1-CD, the minimal functional unit of MUC1, in preneoplasia not only advances our understanding of the molecular basis of MUC1-mediated mammary gland tumorigenesis but may also suggest MUC1-CD as a potential target for the diagnosis and chemoprevention of human breast cancer.

## Materials and Methods

### Ethics statement

All research involving human participants have been approved by the Institutional Review Board of Shanghai Huangpu Center Hospital. Since paraffin-embedded human breast archival specimens were analyzed anonymously, we have not obtained informed consent from all participants involved in this study. All clinical investigation have been conducted according to the principles expressed in the Declaration of Helsinki.

All animal experiments were performed according to protocols approved by the Animal Ethics Committee at the Shanghai Jiaotong University School of Medicine, and their care was in accord with the institution's guidelines (Approval ID: scxk(Hu) 2004-0001).

### Plasmid construction and transgenic mouse generation

A GFP-tagged human cDNA coding MUC1-CD (72 AA) was subcloned into the EcoRI restriction site of the mouse mammary tumor virus long terminal repeat promoter (pMMTV-LTR) transgene cassette [28], [29]. The XhoI fragment carrying the transgene was purified and microinjected into fertilized eggs from C57BL/6JxCBA F1 mice by the Shanghai Research Center for Model Organisms. The genotypes of these mice were identified by PCR analysis of their genomic DNA using the following primers: forward 5′ CTGGTCATCATCCTGCCTTT 3′ and reverse 5′ TTTTGGCAGAGGGAAAAAGA 3′.

### Semiquantitative reverse transcription PCR

Total RNA was isolated from the third mammary glands, salivary gland, lung, ovary and kidney with TRIzol reagent (Invitrogen, Carlsbad, CA) according to the manufacturer's instructions. DNase I-treated (Promega, Madison, WI) total RNA was reverse transcribed with AMV reverse transcriptase (TAKARA Biotechnology, Inc., Otsu, Japan). cDNA was amplified using primers for GFP-CD and β-actin: GFP-CD (forward 5′AAGACCCCAACGAGAAGC 3′ and reverse 5′CTACAAGTTGGCAGAAGTGG 3′); and β-actin (forward 5′ CTGGCCGGGACCTGACAGACTACC 3′ and reverse 5′ATCGGAACCGCTCGTTGCCAATAG 3′). Amplification products were separated by agarose gel electrophoresis and visualized by ethidium bromide staining. The gels were scanned using a Tanon imaging workstation (Tanon, Shanghai, China).

### Quantitative real-time reverse transcription PCR

Quantitative RT-PCR was carried out with SYBR Green PCR kit according to the manufacturer's instructions (Takara Biotechnology, Inc., Otsu, Japan). Amplifications were performed in ABI PRISM 7500 Sequence Detection System (Applied Biosystems, Foster City, CA, USA). Relative transcript quantities were calculated using the ΔΔCt method with β-actin as an endogenous control for normalization. The value of each genotype at each time point was identified by more than three samples and each sample was repeated three times independently. The primer sequences were as following: c-Myc (forward 5′ GTACCTCGTCCGATTCCACG 3′ and reverse 5′ GGGTTTGCCTCTTCTCCACAG 3′); cyclin D1 (forward 5′ GCGTACCCTGACACCAATCTC 3′ and reverse 5′ CTCCTCTTCGCACTTCTGCTC 3′); β-actin (forward 5′ TGTCCACCTTCCAGCAGATGT 3′ and reverse 5′ AGCTCAGTAACAGTCCGCCTAG 3′).

### Immunohistochemistry (IHC)

Mammary gland specimens were fixed in 4% paraformaldehyde in PBS and dehydrated prior to embedding in paraffin. For histological analysis, 6-µm sections were cut and stained with hematoxylin and eosin according to standard procedures. For IHC analysis, paraffin-embedded sections were deparaffinized with xylene and treated with gradually decreasing concentrations of ethanol, then heated at 92–96°C for 30 min in 10 mM citrate buffer (pH 6.0) for antigen retrieval. Sections were treated with 1% hydrogen peroxidase in PBS for 10 min to block endogenous peroxidase activity and blocked for 1 hour in 5% bovine serum followed by staining overnight at 4°C with rabbit anti-GFP (Cell Signaling Technology, Danvers, MA) and rabbit anti-β-catenin antibodies (Santa-Cruz Biotechnology, Inc., Santa-Cruz, CA) for mouse tissues and mouse anti-c-Myc and mouse anti-cyclin D1 antibodies for human specimens. The staining procedure followed the manufacturer's instructions for the ABC staining system (Santa-Cruz Biotechnology).

### Immunofluorescence(IF)

Mammary glands were embedded carefully in O.C.T. Compound (Sakura Finetek USA, inc., Torrance, CA) and stored at -80°C. 6-µm sections were washed with PBS for 5 minutes to remove excess O.C.T. and fixed in freshly prepared 4% paraformaldehyde for 10 minutes at room temperature. The slides were then permeabilized with 0.5% Triton X-100 in PBS for 5 minutes and blocked for 1 hour in 5% goat serum in PBS followed by incubating with primary antibody rabbit anti-GFP (Cell Signaling Technology, Danvers, MA) overnight at 4°C, and fluorescent-conjugated secondary antibody for 30 minutes. Finaly, the slides were rinsed with PBS and mounted with VECTASHIELD-mounting medium (H-1200, Vector Laboratories, Inc., Burlingame, CA).

### Mammary whole-mount staining

Whole-mount staining was performed according to standard protocols (http://mammary.nih.gov/index.html). Briefly, the inguinal mammary glands were harvested and spread on glass slides, fixed overnight in Carnoy's fixative (60% ethanol, 30% chloroform, and 10% glacial acetic acid) at room temperature and washed in 70% ethanol for 15 min, then transferred gradually to distilled water. The slides were stained in carmine alum solution overnight, gradually washed through a series of 70%, 95%, and 100% ethanol for 15 min and cleared in xylene. Images of the whole mounts were acquired with an Olympus digital camera using the same magnification and lighting conditions.

### BrdU staining assay

Mammary cell proliferation was measured by the incorporation of 5-bromo-2′-deoxyUridine (BrdU). BrdU was administered intraperitoneally to mice at a dosage of 5 mg/kg body weight 2 h before sacrifice. BrdU incorporation was detected in sections by IHC staining with Rat anti-BrdU (Santa-Cruz Biotechnology). For each mammary sample, BrdU-positive cells and total mammary epithelium cells were counted in 10 random high-power fields (400×) by an investigator unaware of the animal's genotype. The results are presented as a percentage.

### Western blot analysis

Nuclear and cytoplasmic fractions from mouse mammary gland tissues were prepared using the NE-PER Nuclear and Cytoplasmic Extraction Reagents kit (PIERCE Biotechnology, Rockford, IL) according to the manufacturer's instructions. Mammary protein lysates were prepared as described previously [12]. Proteins were subjected to separation by SDS-PAGE, transferred to nitrocellulose membranes, and probed with rabbit anti-β-catenin, goat anti-lamin B (Santa-Cruz Biotechnology), mouse anti-α-tubulin (Sigma-Aldrich Co., St. Louis, MO), mouse anti-c-Myc, mouse anti-Cyclin D1 and goat anti-β-actin (Santa-Cruz Biotechnology).

### Human breast clinical sample collection

Paraffin-embedded archival specimens were collected from the Department of Breast Surgery, Shanghai Huangpu Central Hospital, China. A total of 11 breast cancer specimens and 11 normal mammary specimens were enrolled in this study, as determined pathologically. These patients did not receive any preoperative adjuvant radiation or chemotherapy.

### Statistical analysis

All quantitative data in this study are presented as mean±standard deviation. A two-tailed Student t-test was used to analyze comparisons between two groups. P<0.05 was considered statistically significant.
